# Beyond the ‘second brain’: the gut microbiota as a constitutive co-constructor of embodied cognitive network

**DOI:** 10.3389/fnins.2026.1808839

**Published:** 2026-06-03

**Authors:** Yue Gou, Xuemei Liu, Wenjie Zhu, Yanling Yuan, Yu Wang, Qinglian Xie

**Affiliations:** 1 Department of Outpatient, West China Xiamen Hospital, West China Hospital, Sichuan University, Xiamen, Fujian, China; 2 Institute of Integrated Traditional Chinese and Western Medicine, West China Hospital, Sichuan University, Chengdu, China; 3 West China School of Medicine, Sichuan University, Chengdu, China; 4 Department of Pharmacy, West China Hospital, Sichuan University, Chengdu, China; 5 Mental Health Center, National Center for Mental Disorders, West China Hospital, Sichuan University, Chengdu, China

**Keywords:** 4E cognition, cognitively active metabolites, embodied cognition, gut microbiota, gut-brain axis, holobiont, psychobiotics, symbiotic co-constructor

## Abstract

Traditional cognitive science has historically confined the mind within the cranium. While the “second brain” metaphor underscores the autonomy of the enteric nervous system, it remains entrenched in a neurocentric paradigm. Here, we propose a transformative framework: the gut microbiota may function as a constitutively relevant contributor to specific embodied cognitive architectures. We contend that cognition, emotion, and behavior are not fully understandable in brain-isolated terms. Instead, these processes emerge from a sustained, bidirectional dialogue between the host and its symbiotic microbial ecosystem. Integrating 4E cognition theory, we systematically delineate how gut microbiota functions as an embedded signaling system—producing cognitively active metabolites, such as short-chain fatty acids and neuroactive substances—to shape interoceptive states and neural function via neural, immune, and metabolic/endocrine interfaces. We establish a rigorous evidential chain, categorized as “deprivation, replacement, observation, and intervention,” synthesizing germ-free animal models, fecal microbiota transplantation, human multi-omics, and clinical interventions. These data—drawn from animal models that establish causal necessity and sufficiency, human cohort studies that reveal systematic ecological associations, and proof-of-concept intervention trials that demonstrate clinical plasticity—converge to support the view that microbiota-derived processes may be constitutively relevant to the realization of specific embodied cognitive architectures, especially those organized through interoceptive prediction, affective appraisal, and vagal-metabolic signaling, rather than functioning as merely transient or incidental regulators. The multi-level nature of this evidence base, spanning causal mechanisms in controlled settings to ecological validity in human populations, provides a robust foundation for reframing the gut microbiota as a symbiotic co-constructor of the embodied mind. Ultimately, we move beyond the linear “gut-brain axis” model to outline a multispecies framework for understanding the embodied architectures within which interoceptive, affective, and related cognitive processes unfold. This paradigm shift offers a novel biological foundation for the mind and enables precision interventions for mental health, such as psychobiotics and targeted ecological remodeling. Looking forward, we envision a unified “microbiota-mind” model that integrates computational modeling and ethical frameworks. This endeavor challenges the traditional concept of a “self” bounded by the skin, providing a roadmap for the future of precision psychiatry and cognitive science.

## 1 Introduction

### 1.1 Gut microbiota: the human holobiont and the biological conditions of cognition

Microorganisms represent the earliest life forms on Earth, and have co-evolved with multicellular organisms over billions of years (Schlebusch et al., 2017; Schopf et al., 2018; Merino et al., 2019; Cheng et al., 2021; Ahmad et al., 2022; Shahab and Shahab, 2022). Through this prolonged co-evolution, microbes have established stable and intimate relationships with their hosts, including humans, profoundly shaping the host’s immune systems and physiological functions (Thaiss et al., 2016; Gupta et al., 2017; Rook et al., 2017; Campbell et al., 2020; Bosco and Noti, 2021).

The human gut constitutes the primary site of this symbiotic interaction, and represents the largest microbial habitat in the body. Approximately 3.8 × 10^13^ microbial cells reside in humans, a number comparable to that of human somatic cells, with the vast majority colonizing the gastrointestinal tract (Sender et al., 2016; Thursby and Juge, 2017). This microbial community comprises bacteria, archaea, fungi, viruses, and protists (Rajilić-Stojanović and de Vos, 2014). The term “gut microbiota” specifically refers to its bacterial component (Ursell et al., 2012). Rather than existing as isolated species, these microorganisms constitute a highly complex structured ecological network characterized by widespread mutualism, competition, and antagonism (Sonnenburg and Sonnenburg, 2019; Drew et al., 2021).

The gut microbiome encodes a gene repertoire that vastly exceeds the human genome, potentially by over two orders of magnitude (Human Microbiome Project Consortium, 2012). Metagenomic studies have uncovered its immense functional capacity; its role as an essential “external” metabolic and regulatory toolbox for the host has become a scientific consensus (Zhang et al., 2025). The gut microbiota influences multiple host systems by generating a wide range of bioactive metabolites, including short-chain fatty acids (SCFAs), neurotransmitter precursors, and secondary bile acids. Through direct interactions with host cells, it plays a key role in regulating immune function, metabolic homeostasis, and neural activity. Furthermore, its functional diversity provides the host with robust buffering and compensatory capacities in response to environmental challenges (e.g., dietary changes and psychological stress) (Yatsunenko et al., 2012; David et al., 2014).

Accumulating evidence further demonstrates that microbial dysbiosis is closely linked to a range of neuropsychiatric disorders, including major depressive disorder (MDD), anxiety disorders, autism spectrum disorder (ASD), Parkinson’s disease (PD), and Alzheimer’s disease (AD) (Warren et al., 2024; Xie et al., 2024; Yu et al., 2024; Zhao et al., 2024; Wang L. et al., 2025; Xu et al., 2025). Taken together, these findings suggest that human neural development and brain function may partially depend on microbial-derived signals, supporting the view of the gut microbiota as a “forgotten organ” (O’Hara and Shanahan, 2006). However, unlike conventional organs, it constitutes a dynamic, transspecies ecosystem composed of trillions of independent microbial life forms. Its functions emerge from continuous dynamic crosstalk and synergy between the host and complex microbial networks rather than fixed anatomical structures, providing a biological foundation for considering cognition as an embodied and distributed process.

### 1.2 Gut microbiota and mental health

Gut dysbiosis refers to significant aberrations in the composition, function, or metabolic activity of the gut microbiota. Such disruptions impair the homeostatic balance between the host and microbes, thereby exerting profound effects on the host’s physiological and psychological states (Lozupone et al., 2012; Carding et al., 2015). Evidence from animal models, human cohort studies, and interventional research consistently indicates that gut dysbiosis is closely associated with a spectrum of neuropsychiatric disorders, suggesting that the gut microbiota may actively contribute to the physiological foundations of emotional regulation, social behavior, and higher cognitive functions via immune, metabolic, and neuroendocrine pathways (McGuinness et al., 2022; Meng et al., 2022).

Patients with depression and anxiety commonly exhibit reduced gut microbiota diversity, characterized by a depletion of anti-inflammatory or SCFA-producing genera (e.g., *Faecalibacterium*) and an expansion of potentially pro-inflammatory genera (Jiang et al., 2015; Zheng et al., 2016; Chen et al., 2024; Guo et al., 2024). Such alterations are associated with decreased SCFA production and elevated levels of pro-inflammatory cytokines. Importantly, dysbiosis drives a shift in the tryptophan metabolism away from serotonin synthesis toward the kynurenine pathway, resulting in imbalanced neuroactive metabolites and exacerbated neuroinflammation (Gao et al., 2020; Lukić et al., 2022). Randomized controlled trials (RCTs) further confirmed that targeted probiotics or prebiotic interventions can partially restore microbial balance and alleviate depressive and anxiety symptoms (Ng et al., 2018). The anxiety-like behaviors observed in germ-free (GF) mice and their reversal following fecal microbiota transplantation (FMT) (Hsiao et al., 2013), provide causal evidence that the microbiome is functionally necessary for normal emotional regulation in these models. These findings converge on a transdiagnostic regulatory axis linking inflammation, SCFA signaling, tryptophan metabolism, and cognition-related processes.

In ASD, gut dysbiosis has emerged as a key pathophysiological feature. Patients typically show dysregulated ratios of Bacteroidetes to Firmicutes, reduced microbial diversity, increased abundance of *Clostridium* and *Ruminococcus*, and decreased levels of beneficial genera such as *Prevotella* and *Bifidobacterium* (Kang et al., 2013; Sharon et al., 2019; Vernocchi et al., 2022). These changes in composition are accompanied by functional metabolic shifts, particularly toward enhanced kynurenine pathway activity and the accumulation of neurotoxic metabolites (Vernocchi et al., 2022; Yu et al., 2024).

Critical evidence derives from FMT studies: transplantation of gut microbiota from ASD donors into GF mice reproduces core behavioral abnormalities, including social deficits and stereotyped behaviors (Sharon et al., 2019). These behavioral phenotypes co-occur with intestinal barrier dysfunction, neuroinflammation, and altered cerebral gene expression, robustly demonstrating that gut dysbiosis may actively shape the neurobiological mechanisms of social cognition (Vernocchi et al., 2022; Feng et al., 2024; Yu et al., 2024).

Gut dysbiosis is also increasingly implicated in neurodegenerative disorders such as AD and PD, where it contributes to disease progression primarily by modulating neuroinflammation and protein misfolding. In PD, patients show a marked depletion of SCFA-producing gut taxa (e.g., *Faecalibacterium prausnitzii*) and an expansion of pro-inflammatory microbiota (Wang L. et al., 2025). Reduced SCFA levels, particularly butyrate, impair intestinal barrier integrity and blood-brain barrier (BBB) function, exacerbating microglia-mediated neuroinflammation. Studies have revealed that *Akkermansia* may attenuate microglial activation and neuroinflammation via regulating butyrate biosynthesis, potentially protecting dopaminergic neurons in the substantia nigra (Xu et al., 2025). A central mechanistic pathway involves gut dysbiosis promoting aberrant α-synuclein aggregation within the enteric nervous system (ENS), followed by retrograde transport to the brain via the vagus nerve (Wang L. et al., 2025). In AD, microbial dysbiosis correlates closely with amyloid-β (Aβ) plaque deposition and neurofibrillary tangle formation (Sampson et al., 2016; Bedarf et al., 2017). Gut microbial metabolites disrupt BBB integrity, trigger neuroinflammation, thereby directly or indirectly accelerating Aβ aggregation and Tau hyperphosphorylation (Warren et al., 2024; Xie et al., 2024). These pathological cascades ultimately impair synaptic plasticity and neuronal dysfunction, manifesting as cognitive deficits in learning, memory, and executive control. Furthermore, the detrimental effects of chronic stress are partly mediated through the microbiota-gut-immune-brain axis, forming a vicious cycle that accelerates cognitive decline (Warren et al., 2024).

Notably, gut-brain communication is bidirectional. Psychological states and central neural activity exert reciprocal regulatory effects on the intestinal environment. Through autonomic nervous system signaling, gastrointestinal motility and secretion are modulated, while activation of the hypothalamic-pituitary-adrenal (HPA) axis releases stress hormones that alter intestinal immune homeostasis and microbial composition. This bidirectional crosstalk establishes a feedback loop in which chronic stress further disrupts microbial homeostasis, thereby reinforcing stress-related neurobiological dysregulation (Fond et al., 2014). Clinically, this bidirectional crosstalk is reflected in the high comorbidity between neuropsychiatric disorders and gastrointestinal conditions, such as the frequent co-occurrence of irritable bowel syndrome (IBS) and mood disorders, which represents a classic model of the “brain-gut” axis in action (Mayer et al., 2015; Pinto-Sanchez et al., 2017). Within this closed feedback loop, the gut microbiota continuously interacts with and co-evolves with the host, highlighting its role in shaping host physiology and cognitive function.

In conclusion, cross-species and multi-modal studies converge to demonstrate that gut microbial alterations are not merely epiphenomena of neuropsychiatric disorders, but actively shape the physiological mechanisms of higher-order functions, especially emotion regulation, interoceptive-affective processing, and forms of social valuation. Together, these findings provide a robust scientific rationale for conceptualizing the gut microbiota as a symbiotic co-constructor of the embodied mind.

### 1.3 Embodied cognition and interoception

A central challenge in evaluating the claim that the gut microbiota constitutes part of the cognitive system lies in clarifying what is meant by “cognition” itself. Historically, the term has been used to encompass neural processing, emotional regulation, behavioral outcomes, and even organism-level physiological regulation—a breadth that, while reflecting the complexity of mental life, risks conceptual ambiguity (Chapey, 1983). To address this, we adopt a dual-layered operational definition aligned with the embodied cognition framework.

At the core layer, cognition refers to information-processing functions that enable organisms to adaptively interact with their environment. These functions include learning, memory, decision-making, prediction, and attentional control—processes that can be operationalized through behavioral tasks and neural correlates (Moon et al., 2020). This core layer captures the classical cognitive science emphasis on computation and representation.

At the extended layer, we recognize that these information-processing functions are inseparably intertwined with emotional regulation, interoceptive states, and motivated behavior. Cognition does not occur in a vacuum; it is always embedded in, and shaped by, the affective and physiological contexts of the organism (Jacob et al., 2023). This expanded view does not conflate cognition with emotion or physiology but acknowledges that, in an embodied framework, cognitive operations are realized within the dynamic integration of these dimensions. Rather, it treats emotional and physiological dynamics as constitutive of the embodied conditions under which particular cognitive operations are realized.

Thus, when we argue that the gut microbiota constitutes part of the cognitive system, we adopt a layered claim. At the strongest and best-supported level, microbial signals contribute constitutively to the interoceptive, affective, and physiological scaffolding within which cognition unfolds. They continuously shape the bodily conditions that influence cognitive flexibility, emotional reactivity, behavioral adaptability, and the tuning of neural processing. At a narrower level of core information-processing functions such as prediction, decision-making, and memory, current evidence suggests that microbial signals are tightly integrated with the neural circuits that implement these functions, although whether this amounts to direct constitutive participation in computation in the strict sense remains a matter for further theoretical and empirical clarification (Clark and Chalmers, 1998; Palacios-Garcia and Parada, 2023).

Embodied cognition represents a fundamental paradigm shift from traditional views. It challenges the notion of the mind as an abstract symbol processor, arguing instead that cognitive processes are not performed by the brain in isolation. Rather, they are rooted in the bodily properties of the organism, its sensorimotor system, and its real-time interactions with the environment.

This perspective has evolved from narrow definition emphasizing the body’s structural constraints on cognition to the broader “4E” theoretical frameworks (embodied, embedded, extended, and enactive) (Varela et al., 1991/2017; Wilson, 2002; Anderson, 2003; Clark, 2008; Rowlands, 2010).

Within this expanded framework, cognition not only relies on bodily morphology and sensory modalities but is also deeply embedded within the individual’s physical and social environments. Cognitive resources can extend to external objects and technological systems (Clark and Chalmers, 1998), and cognition is considered as a continuous process realized through active engagement with the world. This perspective emphasizes that the boundaries of the mind are not confined to the skull but are distributed across multi-layered biological, technological, and social systems (Clark and Chalmers, 1998).

This framework draws inspiration from the phenomenological traditions of Heidegger (1927/2012) and Merleau-Ponty (1945/2015). It has recently gained robust empirical support from neuroscience, particularly through the study of interoception—the brain’s sensing and integration of internal bodily states. Research shows that visceral signals (e.g., heart rate, respiration, gastrointestinal activity) are continuously transmitted to the insular cortex, where they fundamentally shape emotional experience, self-perception, and decision-making (Craig, 2002, 2009; Critchley and Garfinkel, 2018). This aligns with the theory of constructed emotion, which proposes that the brain actively constructs emotions by integrating interoceptive inputs with predictive models of the environment (Barrett, 2017).

These findings expand the concept of the “body” in cognitive science. First, the body includes not only the motor limbs and superficial sensory organs, but also deep physiological processes, particularly the visceral environment profoundly shaped by the gut microbiota. Second, studies linking heart rate variability (HRV) and electrogastrography (EGG) to cognitive functions have shown that the visceral signals provides a continuous stream of cognitive information (Giuseppe Forte, 2024; Forte and Casagrande, 2025; Cernanova Krohova et al., 2025). Together, these observations support the theoretical framework of embodied cognition, which regards the gut and its symbiotic microbial ecosystem as the deep physiological foundation of the mind.

### 1.4 Gut microbiota as a symbiotic co-constructor

The “second brain” metaphor highlights ENS autonomy (Gershon, 1998) but remains neurocentric, obscuring the gut microbiota as the key protagonist in gut-brain communication. The ENS serves as a key pathway for signal integration and transmission, but it is not the main source of signaling molecules. To clarify the distinction between the two, Table 1 compares them across five critical dimensions:

**TABLE 1:** Comparison of the enteric nervous system (ENS) and gut microbiota.

Dimension	Enteric nervous system (ENS)	Gut microbiota
Signal source	Intracellular electrochemical activity; encoded by host genes	Extracellular metabolic synthesis; encoded by microbial genomes
Primary mediators	Neurotransmitters (ACh, 5-HT, NO, etc.)	Short-chain fatty acids (SCFAs), GABA, indoles, immunoregulatory factors
Plasticity	Relatively stable after maturation; functional plasticity	Highly dynamic community structure and metabolism; rapidly reshaped by diet, drugs, and stress
Evolutionary level	Neural network of metazoans	Early branches of life (bacteria, archaea); over 1 billion years of coevolution with eukaryotic hosts
Functional role	Signal integrator and transmitter	Signal producer and regulator

In contrast to the relatively stable ENS, the gut microbiota functions as a dynamic “biochemical factory,” producing most key signaling molecules that directly regulate neural activity (e.g., SCFAs, GABA, neurotransmitter precursors) (Cryan et al., 2019). These metabolites form the material basis for gut-brain communication, continuously shaping the visceral states and neurobiochemical environments that influence cognition through immune, endocrine, vagal, and metabolic pathways (Mayer et al., 2015).

This shaping occurs throughout life. In early life, the microbiota influences the development of the HPA axis and sets the threshold for stress responses (Sudo et al., 2004; Li X. et al., 2023), thereby establishing a physiological foundation for lifelong emotional and behavioral patterns (Vogel et al., 2020). In adulthood, its high sensitivity to environment factors (e.g., diet, medication, and stress) allows it to continuously reshape the neuro-immune-metabolic networks that support cognition (Braniste et al., 2014).

The host’s mental states (e.g., stress, emotion) can top-down regulate microbial composition, while the microbiota can actively influence brain function and behavior via bottom-up mechanisms. Specific embodied cognitive architectures emerge, in part, from this cross-species dynamic, perfectly embodying the principles of distributed cognitive processes (Hutchins, 1995). Thus, microbial metabolites are far from passive byproducts of physiological activity but should be regarded as highly integrated physiological signaling resources provided by symbiotic microbes. As a symbiotic system with an independent genome, the microbiota can actively sense the host’s physiological state—such as blood glucose levels and inflammatory signals—and autonomously adjusts its metabolic profile accordingly. Through cognitively relevant metabolites (CAMs), the microbiota serves as a constitutively relevant contributor to the interoceptive, affective, and vagal-metabolic architectures that constrain and tune the brain’s predictive processing, thereby participating in the embodied architectures of interoceptive prediction, affective appraisal, and vagal-metabolic cognition. For instance, during low glucose, it increases propionate synthesis to promote gluconeogenesis. This active sensing-responding-regulating capacity makes the microbiota a dynamically integrated component of the embodied host–microbe system rather than a passive external tool. Unlike the relatively passive notebook-style extension proposed by Clark and Chalmers (1998), the microbiota exhibits an unusually high degree of integration with host systems.

In summary, the gut microbiota goes beyond the neurocentric “second brain” metaphor. It is not a passive background or peripheral modifier of cognitive processes but a “symbiotic co-constructor” that is deeply embedded in and co-constructs neurocognitive functions through its unique genetic, metabolic, and ecological properties. A comprehensive view of the gut-brain axis should consider a multispecies, multi-layered embodied cognitive network. Within this network, the mind is neither solely generated by the brain nor independently determined by the microbiota, but it emerges from the continuous dynamic interaction between the two. Emphasizing the microbiota as a “co-constructor” highlights the complete biological basis of mental processes, without diminishing the brain as the ultimate hub for information integration and conscious experience. Mechanistically, the regulatory role of microbial metabolites at the molecular level may help explain how microbial processes become deeply embedded in the systemic architecture within which cognition unfolds.

### 1.5 Criteria for constitutiveness and the boundaries of the cognitive system

If the gut microbiota is to be considered a constitutive component of the cognitive system, a principled distinction must be drawn between elements that belong to the system and those that merely influence it from within (e.g., hormones, cytokines) or from without (e.g., light, temperature, social interaction). To address this, we propose five intersecting criteria that collectively define what it means for an element to be a constitutive component of a cognitive system. These criteria, derived from philosophical accounts of cognitive systems (Rupert, 2009; Kaplan, 2012), and empirical studies of the microbiota–gut–brain axis, allow us to distinguish constitutive participation from mere modulation or environmental influence.

Criterion 1: Spatial location. Constitutive components are instantiated within the organism’s physical boundaries, forming part of the organism’s internal physiological milieu. The microbiota resides in the gastrointestinal tract, constituting a permanent feature of the internal milieu (Sender et al., 2016; Thursby and Juge, 2017). Hormones and cytokines also originate internally and thus satisfy this criterion, whereas environmental factors such as light, temperature, and social interaction are external to the body and do not.

Criterion 2: Temporal continuity. Constitutive components maintain a persistent presence, providing a continuous background signal stream against which transient perturbations occur. The microbiota is continuously present from birth, establishing a stable ecological community that fluctuates within a homeodynamic range (Yatsunenko et al., 2012; David et al., 2014). Many hormones and cytokines are often released in pulses or in response to discrete events; they function as transient signals rather than ongoing constitutive conditions. Environmental factors are inherently intermittent, constituting discrete events rather than continuous background conditions (Clark and Chalmers, 1998).

Criterion 3: Functional integration. Constitutive components are woven into the functional architecture of the system, participating in multiple regulatory circuits simultaneously. The microbiota is not a single signal but an ecosystem whose metabolic outputs are integrated into host physiology across immune, endocrine, and neural systems (Shoubridge et al., 2022; Malan-Mueller et al., 2025). It operates as a functional part of the organism’s regulatory infrastructure. Hormones and cytokines are more often discussed within specific signaling axes, whereas the microbiota is distinctive in operating as an ecologically organized, metabolically generative subsystem with outputs distributed across multiple host interfaces (Palacios-Garcia and Parada, 2023). Environmental factors exert influence through dedicated sensory pathways without being integrated into the system’s internal regulatory networks.

Criterion 4: Participation in cognitive architecture. Constitutive elements need not always implement computation in the same way as neurons, but they must make an ongoing, non-incidental contribution to the organization of the physiological architecture within which cognitive processing is realized. This criterion therefore concerns constitutive organization of cognitive architecture rather than neural implementation alone. In this sense, microbial metabolites such as short-chain fatty acids, tryptophan-derived compounds, and GABA-related products shape synaptic plasticity, neural oscillatory regimes, network connectivity, and interoceptive signaling states that are integral to cognitive processing. We therefore regard the microbiota as contributing constitutively to the embodied architecture of cognition. At the same time, we acknowledge that whether such contributions should be described as direct participation in computation in the narrowest sense remains theoretically contested.

By contrast, hormones and cytokines are also deeply relevant to cognition and may in some contexts play constitutive roles of their own. Our point is not that these systems are merely incidental, but that the microbiota is distinctive as a continuously present, cross-species, metabolically generative subsystem whose outputs are distributed across neural, immune, and endocrine pathways simultaneously.

Criterion 5: Developmental embedding. Constitutive components are indispensable for the normal structural development of the system. Germ-free animals exhibit lasting, often irreversible deficits in neurogenesis, blood–brain barrier integrity, and stress response systems—deficits that reflect qualitative alterations in the neural substrates of cognition (Sudo et al., 2004; Braniste et al., 2014; Ogbonnaya et al., 2015). More recent work has extended these findings by showing that even subtle, naturally occurring differences in the gut microbiome during early life can shape behavioral outcomes (McAdams et al., 2024), and that the maternal microbiome exerts lasting effects on fetal neurodevelopment (Di Gesu and Buffington, 2024). Moreover, microbial metabolites directly influence neuronal structure during critical windows: in a genetic model of depression, early-life reduction of *Akkermansia muciniphila* was associated with reduced dendritic arborization and spine density, and supplementation reversed these structural deficits (Hwang et al., 2025). These findings indicate that the microbiota is not merely modulatory but actively shapes neural architecture during development, fulfilling the criterion of developmental indispensability.

No single criterion alone suffices to establish constitutive status, and we do not claim that the microbiota is the only non-neural contributor relevant to cognition. Rather, our argument is that the microbiota is distinctive in the convergence of multiple features: persistent internal presence, cross-system functional integration, developmental embedding, and ongoing shaping of the interoceptive and physiological architecture within which cognition is enacted. These criteria do not eliminate all boundary disputes, but they provide a structured basis for regarding the microbiota as a constitutively relevant contributor to the interoceptive, affective, and vagal-metabolic architectures of cognition—rather than as a merely transient regulator or external influence.

A note on distinguishing evidence: hormones and cytokines also influence cognition. However, recent studies of depression-associated cognitive impairment and vagus-dependent working-memory deficits suggest that microbial signals may explain task-specific variance not reducible to global inflammatory or endocrine state changes (Jia et al., 2024; Yue et al., 2026). Future research directly comparing matched manipulations of microbial versus hormonal pathways would further clarify the distinctive constitutive contribution of the microbiota.

### 1.6 What counts as constitutive rather than merely causal?

A central challenge in evaluating constitutive claims is distinguishing between causal influence and constitutive relevance. A factor may influence a system without being part of its constitutive architecture (Rupert, 2009). To avoid the coupling–constitution fallacy, we adopt a mutual manipulability criterion (Kaplan, 2012; Krickel, 2018): a component is constitutively relevant to a system-level phenomenon if (1) intervening on the component changes the phenomenon, and (2) intervening on the phenomenon (via other components) changes the component’s state in a systematic, non-incidental manner.

Constitutive relevance does not require the component to be neural or to operate at millisecond timescales, but it does require mechanistic integration—an identifiable role within the causal structure realizing the phenomenon—and that its contribution should be ongoing and not readily substitutable without altering the realization of the target cognitive process.

Applying this criterion, we do not claim that the microbiota is a constitutive part of all cognitive processes. Rather, for specific embodied cognitive architectures—particularly those organized through interoceptive prediction, affective appraisal, and vagal–metabolic signaling—microbiota-derived signals meet the mutual manipulability condition: manipulating the microbiota alters interoceptive and affective processing, and manipulating those interoceptive or affective states (e.g., via stress or reward-related paradigms) systematically alters microbial composition. Here, the point is not that any downstream host-state change suffices, but that these states participate in a reciprocal host–microbe loop organized around the same embodied cognitive architecture.

## 2 The embodied cognition network: gut-brain axis–gut microbiota–host

### 2.1 The gut microbiota: the microbial dimension of the embodied mind

Within the framework of embodied cognition, humans can be conceptualized as a “holobiont”—a symbiotic superorganism—whose physiological and psychological functions emerge from long-term coevolutionary interactions between the host and the microbiota (Margulis and Fester, 1991; Bordenstein and Theis, 2015). These cross-species interactions enable environmental factors (e.g., diet) to be biologically encoded into host signaling pathways, thereby shaping neural activity related to emotion, motivation, and decision-making. This perspective challenges traditional views that confine cognition exclusively to the brain.

From this perspective, the gut microbiota may be considered an embedded symbiotic metabolic signaling system that continuously produces neuroactive and immunomodulatory molecules shaping host physiology. Supported by dietary substrates provided by the host, microbial metabolism generates a repertoire of neuroactive and immunomodulatory molecules that contribute to the maintenance of physiological and cognitive homeostasis (Cerdo et al., 2016; Chen et al., 2025; Cichon et al., 2025).

For example: (1) SCFAs—including butyrate, propionate, and acetate—are not only energy sources for colonocytes but also potent epigenetic modulators that influence microglial maturation, blood-brain barrier (BBB) integrity, and hippocampal neurogenesis and plasticity (Stilling et al., 2016; Dalile et al., 2019). (2) Neurotransmitter-related metabolism. Although over 90% of serotonin (5-HT) is synthesized by enterochromaffin cells, the gut microbiota critically regulates its bioavailability by modulating tryptophan metabolism (kynurenine pathway and serotonin pathway) (Clarke et al., 2013). (3) Other bioactive metabolites. Specific Lactobacillus and Bifidobacterium strains can directly synthesize γ-aminobutyric acid (GABA) or its precursors (Strandwitz, 2018). Additionally, microbiota-derived bile acids, histamine, lipopolysaccharide (LPS), and bacterial extracellular vesicles (EVs) all modulate host immune and neural function (Díaz-Garrido et al., 2021). Together, these metabolic processes form a highly integrated microbiota-host co-metabolism network. Microbial biochemical activity depends on host-provided environmental conditions, while microbial metabolites reciprocally regulate the host’s immune, endocrine, and nervous system states (Cryan and Dinan, 2012). Notably, this regulation is bidirectional communication rather than strictly top-down, with convergence occurring in the central nervous system via neural (e.g., vagus nerve), immune (e.g., cytokines), and endocrine (e.g., gut peptide hormones) pathways.

Thus, microbial metabolites can be regarded as a form of quasi-interoceptive input: although originating from non-self microbial life, they provide the brain with continuous, dynamic stream of physiological information about the body’s internal state (e.g., energy availability and immune activation levels). This microbial signaling stream integrates with traditional interoceptive signals from host organs and participates in the construction of interoceptive-affective states, stress responses, and forms of social valuation, thereby forming an important and deeply integrated signaling layer of the embodied mind (Ntiri and Wong, 2025).

### 2.2 The gut microbiota: signal interfaces of the embodied cognition network

**FIGURE 1:**
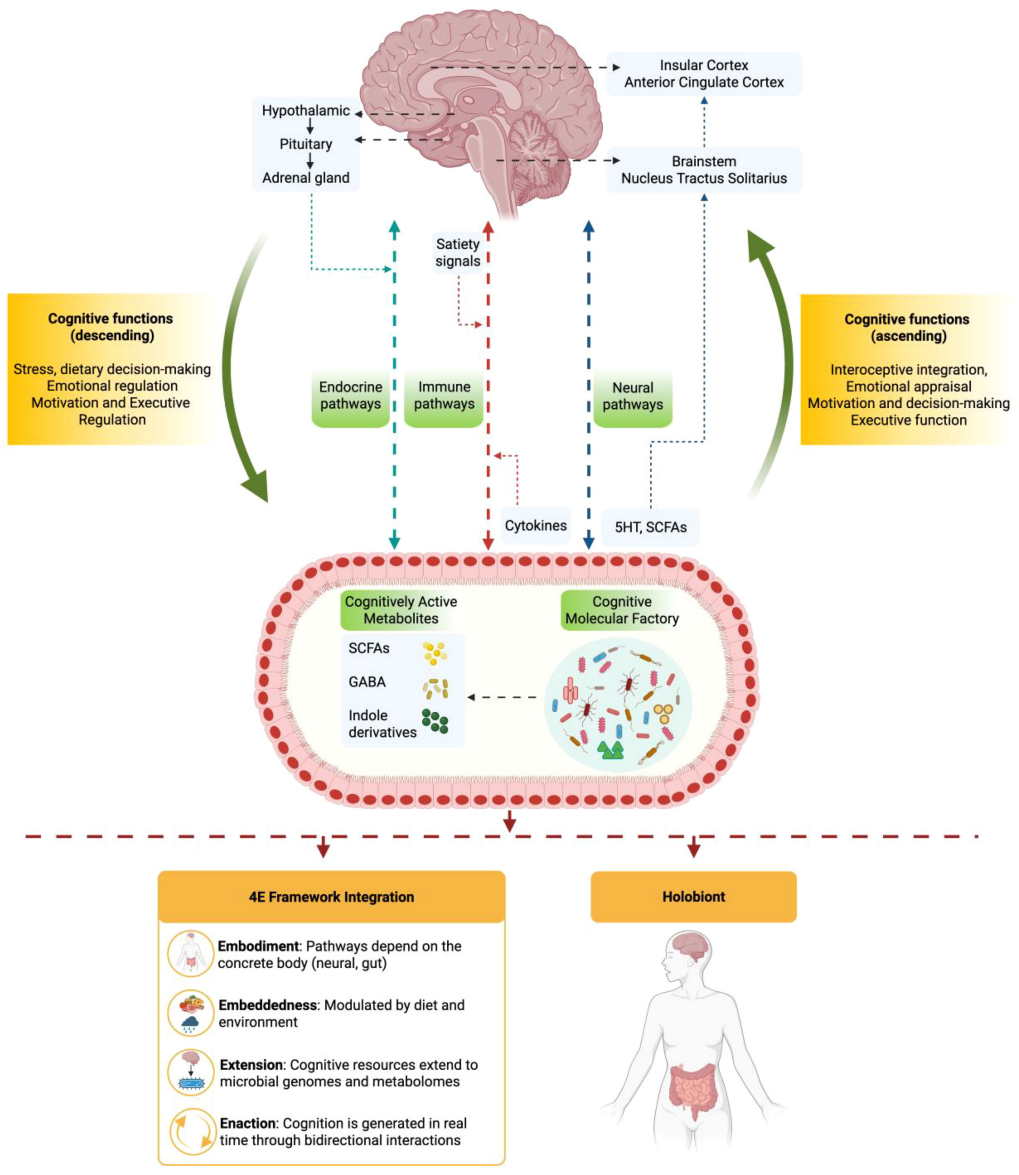
Microbes as symbiotic cognitive co-constructors: a model of embodied, distributed cognition. This schematic illustrates the core theoretical framework: the gut microbiota acts as a symbiotic co-constructor within an embodied, distributed cognitive network. Core entities: The holobiont and biosynthetic hub. The human host and gut microbiota form an integrated “host-microbe holobiont” as the fundamental unit of cognition. The microbiota functions as an embedded metabolic signaling system, continuously producing key CAMs (e.g., SCFAs, GABA, indoles) which form the material basis of ascending signaling flux. Three embodied interfaces: Ascending signaling pathways. (1) Neural Interface (blue): centered on the vagus nerve, transmitting gut-derived signals (e.g., 5-HT, SCFAs) to the brainstem nucleus tractus solitarius (NTS), which subsequently projects to the insula and anterior cingulate cortex (ACC) for interoceptive integration and emotional appraisal. (2) Immune interface (red): Microbial metabolites regulate intestinal immune cells to regulate cytokine release or directly influence blood-brain barrier (BBB) integrity and microglial function, thereby maintaining neuroimmune homeostasis. (3) Metabolic/endocrine interface (green): The microbiota influences enteroendocrine cells to secrete hormones (e.g., GLP-1, PYY), which exert systemic effects on the hypothalamic-reward-prefrontal circuitry via circulation, regulating energy balance, motivation, and executive control. Foundational system properties. (1) Bidirectionality: All pathways are bidirectional. Descending arrows represent top-down modulation of the gut environment and microbiota by the brain via neural and endocrine outputs. (2) Dynamic plasticity: Dynamic circular motifs symbolize continuous activity and adaptive plasticity. Microbial composition and network connectivity exhibit high remodeling capacity in response to diet, stress, and interventions. 4E framework integration: Embodiment: Pathways depend on the concrete physiological structures (neural, gut); Embeddedness: Function is modulated by diet and environment; Extension: Cognitive resources extend to microbial genomes and metabolomes; Enaction: Cognition is generated in real time through bidirectional interactions.

As shown in Figure 1, interactions between the gut microbiota and the central nervous system (CNS) form a cross-species, multi-layered embodied cognition network that aligns closely with the 4E cognitive framework. The vagus nerve and ENS provide the anatomical basis for embodiment, enabling microbe-derived signals to directly access central processing circuits (Breit et al., 2018; Kaelberer et al., 2018). Signal intensity and composition are modulated by diet and social context, reflecting embeddedness (Cryan et al., 2019). Microbial metabolites, as exogenous yet physiologically integrated molecules, contribute to the embodied conditions under which neural processing is organized, thereby extending the biological architecture relevant to mental functions (Dalile et al., 2019). The interactive nature of this system is illustrated by stress-related feedback loops. For example, sympathetic nerve activation alters intestinal motility and luminal pH, thereby inducing the rapid upregulation of SCFA-related gene expression (e.g., but genes). These SCFAs are transmitted to the nucleus tractus solitarius (NTS) via the vagus nerve, where they modulate excessive activation of the prefrontal cortex (PFC) and reduce anxiety-like behaviors. This dynamic closed-loop, host neural signals → microbial metabolic adjustments → neural circuit regulation → cognitive output, illustrates the microbiota’s role in the real-time modulation of cognitive-affective states. In parallel, the host’s shaping of the microbial ecosystem through diet and behavior forms an active cognition-action loop (Foster and McVey Neufeld, 2013).

Based on their functional roles, the network’s signaling pathways can be organized into three interfaces: (1) Neural interface, centered on interoceptive input. This interface integrates mechanical and chemical gut signals, vagus afferents, enterochromaffin cell-derived serotonin (5-HT), and microbiota-derived metabolites (e.g., SCFAs and GABA) converge in the NTS and are transmitted to the insula and anterior cingulate cortex (ACC), participating in emotional regulation, risk assessment, and attentional control (Bonaz et al., 2018; Kaelberer et al., 2018; Azzalini et al., 2019). The effects of SCFAs on this pathway extend beyond vagal signaling. Butyrate acts as a histone deacetylase inhibitor, increasing acetylation at the *Bdnf* promoter and thereby promoting neurogenesis and synaptic plasticity within the ACC and insula (Nohesara et al., 2023). It also activates free fatty acid receptors (FFAR2 and FFAR3) expressed on neurons and glia, modulating long-term potentiation and network oscillations that underlie attentional control and emotional appraisal (Dalile et al., 2019). Dysregulation of this pathway is directly linked to mood disorders: reduced butyrate correlates with decreased *Bdnf* expression and depressive-like behaviors, effects reversible by butyrate supplementation. Direct evidence for online vagal activation comes from recent work showing that specific microbial metabolites rapidly activate vagal afferent neurons in a receptor-dependent manner, influencing brainstem responses within seconds to minutes (Jameson et al., 2025). Such fast signaling strengthens the plausibility of constitutive participation in ongoing interoceptive-affective operations, rather than merely setting slow background conditions. (2) Immune interface, centered on inflammatory modulation. Microbiota regulation of intestinal immune cells (e.g., macrophages, dendritic cells) shapes systemic cytokine balance, thereby modulating central neuroinflammatory states (Belkaid and Hand, 2014). These immune signals reach the brain either by crossing the BBB or via vagus pathways, further modulating microglial function, synaptic plasticity, and stress response patterns (Erny et al., 2015). Among these, SCFAs play a pivotal role in maintaining microglial development and inhibiting neuroinflammation, as described in the SCFAs–microglia model proposed by Cao et al. (2025), Loh et al. (2024), and Cao et al. (2025). (3) Endocrine and metabolic interface, centered on systemic integration. The gut microbiota regulates hormone release from enteroendocrine cells, such as glucagon-like peptide-1 (GLP-1), peptide YY (PYY), and cholecystokinin (CCK). These signals influence the hypothalamic-reward-prefrontal network through the circulatory system or neural reflexes, thereby regulating energy balance, motivational intensity, and executive control (van de Wouw et al., 2018). Beyond these hormonal pathways, the microbiota shapes cognitive function through tryptophan metabolism. Tryptophan is metabolized by gut bacteria into indole derivatives, which serve as endogenous ligands for the aryl hydrocarbon receptor (AhR)—a transcription factor expressed on immune cells and neurons. AhR activation regulates the expression of inflammatory genes and influences microglial activity, thereby modulating neuroinflammation and synaptic plasticity (Wang et al., 2024). In drug-naïve first-episode schizophrenia patients, aberrant tryptophan metabolism has been identified as a central hub of microbiome-gut-brain crosstalk, linking GABAergic dysfunction to neuroinflammation (Wang et al., 2024). This pathway exemplifies how microbial metabolites can directly influence neurotransmitter systems and inflammatory states through receptor-mediated mechanisms, complementing the hormonal signaling already described. Furthermore, microbiota-derived bile acid and EVs contribute to shaping central metabolic and inflammatory microenvironment in the long term (Sun et al., 2018). A recent review systematically summarizes how SCFAs maintain gut-brain axis homeostasis through multi-layered mechanisms (e.g., barrier preservation, regulation of neurogenesis and synaptic plasticity), providing the latest insights into microbiota-host metabolic integration (Pang et al., 2026).

The three major signaling interfaces do not operate independently but form an integrated immune-endocrine-neural network. For example, SCFAs can activate intestinal dendritic cells to release IL-10 (immune interface), which further inhibits pro-inflammatory enteroendocrine signaling and reduces hypothalamic CRH neurons activation (endocrine interface). Simultaneously, SCFAs directly inhibit locus coeruleus (LC) norepinephrine release via the vagus pathways (neural interface). Together, these three processes downregulate stress responses and achieve homeostatic regulation of cognitive states.

In summary, these three interfaces demonstrate that signal exchange between the gut microbiota and the host is not a passive physiological background process. Instead, it is a dynamic system that actively shapes and maintains the embodied cognitive architecture. Microbial chemical signals are integrated into the embodied and interoceptive conditions of predictive processing, thereby contributing to a distributed host–microbe architecture within which cognition unfolds (Allen and Friston, 2018).

### 2.3 Gut microbiota-brain communication in the framework of embodied cognition

Embodied cognition theory conceptualizes the gut microbiota-host system as a real-time, distributed architecture for embodied cognition. Within this network, the microbial genomic and metabolic activities are tightly integrated with host neural, immune, and endocrine pathways. These cross-species interactions synergistically modulate higher-order functions, such as stress regulation, emotional appraisal, and social motivation, through multi-layered parallel signaling streams (Palacios-Garcia and Parada, 2023; Kohl, 2025). Accordingly, cognition may be understood not as brain-bound in a narrow sense, but as unfolding within a distributed biological system whose neural implementation is continuously shaped by cross-species physiological interactions, rather than an isolated computation to the brain. The core characteristics of this network can be further elucidated through the 4E framework of embodied cognition (Figure 1).

As a dynamic regulator of the internal environment, the gut microbiota continuously releases a diverse array of signaling molecules, including inflammatory mediators, SCFAs, and tryptophan metabolites (Cichon et al., 2025). These signals ascend to the CNS via enteric, vagus, and circulatory pathways, providing persistent interoceptive input that sets the baseline response threshold for emotional and intuitive processes (Loh et al., 2024; Aziz-Zadeh et al., 2025). A specific subset of these molecules, termed CAMs, can cross biological barriers to serve as key mediators of this embodied chemical signaling. For instance, butyrate promotes the expression of hippocampal brain-derived neurotrophic factor (*BDNF*) by inhibiting histone deacetylases (HDACs), while indole derivatives regulate emotional encoding patterns in the amygdala via the AhR–PACAP axis (Rajbhandari et al., 2023; Molska et al., 2024).

This network exhibits remarkable bidirectional plasticity and resilience. Studies have observed that experimental depletion of microbial diversity (e.g., via antibiotics or high-sugar diets) reduces θ-γ wave coupling in the prefrontal cortex within 72 hours, accompanied by the onset of anxiety-like behaviors. Conversely, interventions restoring microbial diversity can re-establish the functional connectivity of the prefrontal-limbic circuit under chronic stress (Jang et al., 2021; Zhu et al., 2025). These findings indicate that emotional regulation and stress resilience reflect the functional integrity of the entire microbiota-host symbiotic system.

Given the sensitivity of microbial composition to diet, hormones, and social context, each individual exhibits a unique “microbial fingerprint.” This highly plastic microbial dimension provides a modifiable interface for embodied cognition. This means that intervention strategies such as precision nutrition, prebiotics/probiotics, targeted FMT, or CAM-based therapeutics can be regarded as system-level recalibrations. By reshaping the interoceptive signaling streams to the brain, these approaches provide new avenues for the personalized regulation of emotion and behavior (Ritz et al., 2024).

Taken together, these insights challenge the traditional philosophical concept of the “self” bounded by the skin or blood-brain barrier. Instead, the self may be better understood as emerging from an extended embodied system that continuously exchanges information and matter with the environment. Within this framework, the gut microbiota plays a dual role in this context: it is both a foreign “other” and a deeply embedded contributor to the embodied organization of cognition. This perspective not only provides a solid biological foundation for the 4E cognitive theory but also raises novel ethical questions regarding the extent to which intentional modification of microbial ecosystems constitutes legitimate “self-modification” (Clark and Chalmers, 1998; Palacios-Garcia and Parada, 2023)?

### 2.4 The strongest case: interoceptive and predictive architectures

The constitutive relevance of the gut microbiota is not uniform across all cognitive domains. The strongest evidence converges on interoceptive, affective, and vagal-metabolic architectures—cognitive processes that rely on real-time signaling of internal bodily states, predictive models of physiological needs, and reward- or threat-related valuation. These domains are especially important because they satisfy the mechanistic conditions outlined in section 1.6 more plausibly than abstract, slower, or more weakly embodied forms of cognition.

First, interoceptive prediction is a core component of embodied cognition. Visceral signals, including those from the gut, are integrated into predictive coding loops that generate emotional experience, action selection, and self-awareness (Craig, 2009; Barrett, 2017). Recent GI interoception reviews explicitly position gut-derived signals as online contributors to reward, affective processing, and decision-making, rather than as modulators of general arousal or metabolic background (Alhadeff and Yapici, 2024; Chang et al., 2025; Salvador et al., 2026).

Second, vagal-metabolic signaling provides a direct, fast pathway. Microbial products such as short-chain fatty acids and indole derivatives activate vagal afferent neurons within seconds to minutes, triggering brainstem and limbic responses that modulate working memory, cognitive flexibility, and stress resilience (Jameson et al., 2025; Yue et al., 2026). This timescale is compatible with online participation in cognitive operations.

Third, affective appraisal—rapid evaluation of stimuli as good/bad, safe/dangerous—is constitutively shaped by interoceptive signals. Microbiota manipulations alter anxiety-like behavior, social valuation, and reward sensitivity in ways not reducible to general sickness behavior. In studies where selective vagal blockade abolishes microbe-to-behavior effects, the resulting pattern is more compatible with a constitutive rather than a purely modulatory interpretation.

By contrast, we do not claim that microbiota are constitutive of abstract reasoning, language, or episodic memory in the same sense. This graded view allows a strong, defensible claim where evidence is most robust, while remaining open to future discoveries.

## 3 Microbes as cognitive co-constructors: an evidential chain from causal intervention to ecological association

**FIGURE 2:**
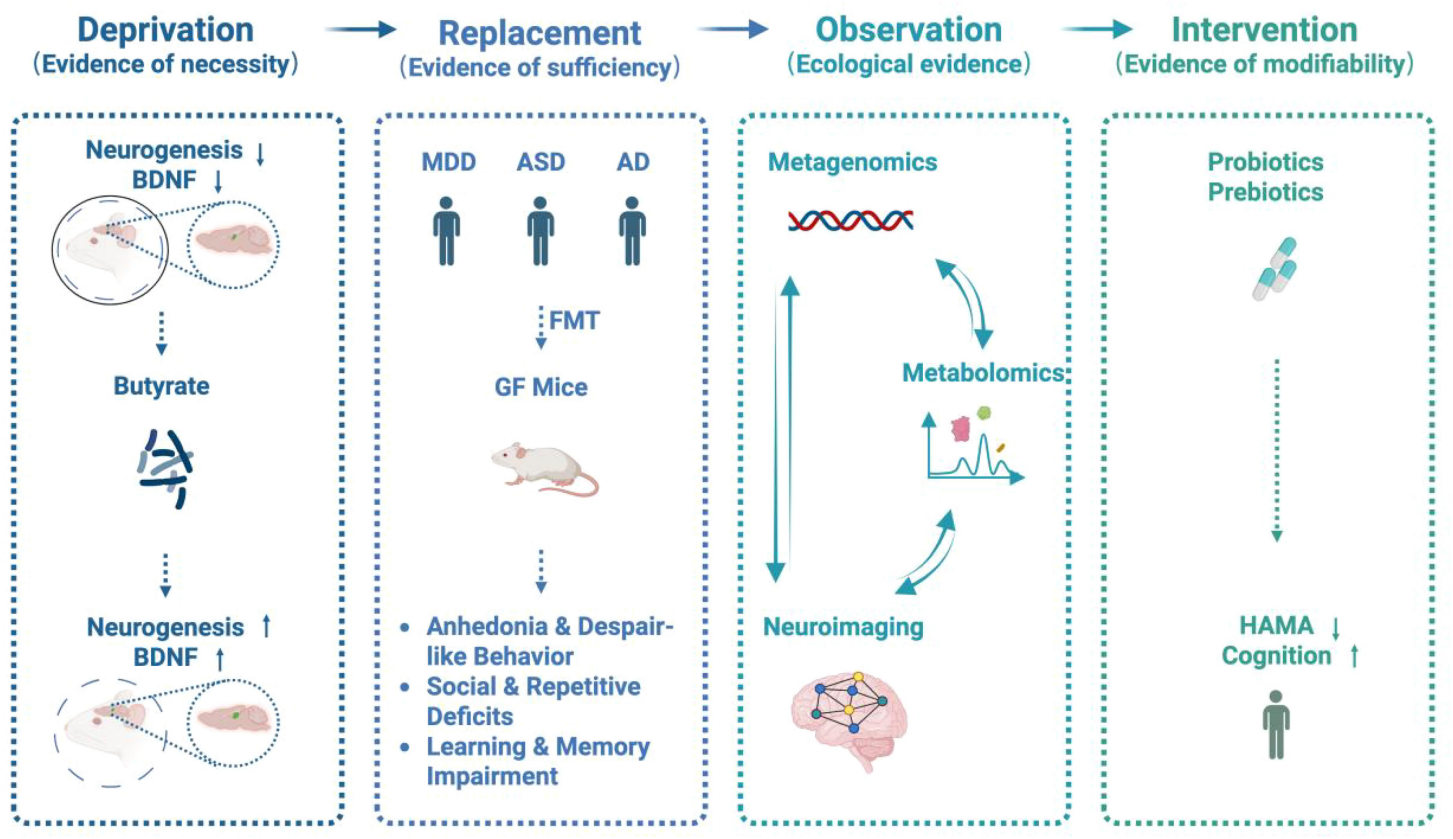
The multi-dimensional evidence chain for the microbiota as a cognitive co-constructor. This figure synthesizes four progressive lines of evidence, ranging from causal inference to population-level association and clinical intervention, to support the role of the gut microbiota within the embodied cognitive network. Evidence structure: a logically progressive framework. The argumentative chain is organized into a four-stage methodological cycle: deprivation → replacement → observation → intervention, representing a methodological transition from controlled experimentation to population-level observation and, ultimately, to clinical intervention. The convergence of these complementary pathways provides a robust empirical foundation for the distributed cognition model. Detailed description of the four evidential pillars. (1) Deprivation (establishing causal necessity): This stage uses GF models or antibiotic treatment to eliminate microbial signals. Findings: Significant impairments are observed in hippocampal-dependent learning, social cognition, emotional behaviors, and neural plasticity mechanisms (e.g., attenuated neurogenesis, reduced *BDNF* expression). Conclusion: These data establish the causal necessity of microbial signals for the normal maturation and maintenance of cognitive architectures. (2) Replacement (demonstrating causal sufficiency). This stage employs FMT to transfer microbial communities from human donors (e.g., MDD, ASD, or AD) into GF or microbiota-depleted recipients. Findings: Recipient animals recapitulate the donor’s behavioral phenotypes (e.g., anhedonia, social deficits) and associated neuropathological features. Conclusion: These studies demonstrate that the microbiota acts as a functional consortium sufficient to drive specific cognitive-behavioral phenotypes. (3) Observation (mapping ecological coupling). This stage involves large-scale human cohort studies integrating multi-omics (e.g., metagenomics, metabolomics) with multimodal neuroimaging. Findings: Significant associations exist between specific microbial abundances and the functional connectivity of large-scale brain networks (e.g., the DMN). Conclusion: Provides ecological evidence for systemic coupling between microbial ecology and higher-order brain functions in natural human populations. (4) Intervention (validating clinical plasticity). This stage focuses on targeted microbial interventions by probiotics, prebiotics, or dietary regimens in randomized controlled trials (RCTs). Findings: Measurable improvements in cognitive performance (e.g., working memory) and emotional states are observed, accompanied by the normalization of peripheral and central biomarkers (e.g., butyrate levels, prefrontal-amygdala connectivity). Conclusion: Confirms the modifiability and clinical plasticity of the system, supporting the microbiota’s role as an active and clinically tractable contributor to the embodied architectures within which specific cognitive processes unfold. Collectively, these four complementary evidential pathways converge to support the core proposition that the gut microbiota acts as an indispensable, active, and clinically tractable cognitive co-constructor.

The core proposition of the “microbiota-host distributed cognitive network” framework is that specific embodied cognitive functions may emerge from dynamic systems jointly organized by central neural activity and microbial signaling. To evaluate this proposition, we construct a multi-dimensional evidence chain, in which each dimension addresses a distinct question about the nature of microbial involvement in cognition (Figure 2).

### 3.1 Deprivation: functional loss experiments establish causal necessity

Deprivation studies—using germ-free animals or antibiotic treatment—ask whether microbial signals are necessary for normal cognitive architecture. GF animal models have shown that the absence of microbe-derived signals significantly impairs the expression of *BDNF* and attenuates neurogenesis in the dentate gyrus, leading to deficits in exploratory behavior and altered anxiety-like behaviors (Delgado-Ocaña and Cuesta, 2024; Nicolas et al., 2024). Furthermore, restoring microbial signals—either via colonization with high butyrate-producing strains or exogenous administration—can rapidly rescue functional state of prefrontal neurons function (e.g., alleviating ferroptosis and restoring electrophysiological activity) and improve cognitive and emotional behaviors (Silva et al., 2020; Cao et al., 2025; Wang Z. et al., 2025). These experiments consistently show that the absence of microbiota leads to structural deficits in neurogenesis, blood–brain barrier integrity, and stress response systems (Sudo et al., 2004; Braniste et al., 2014; Ogbonnaya et al., 2015). More recent work has extended these findings by demonstrating that antibiotic-induced microbial disruption impairs memory plasticity in non-mammalian models, suggesting that the necessity of microbial signals is conserved across species (Davidson et al., 2024). This dimension establishes necessity.

### 3.2 Replacement: FMT enables phenotypic transfer

Replacement studies-fecal microbiota transplantation from patients to animals-ask whether the microbiota is sufficient to drive behavioral phenotypes. FMT studies provide further causal evidence through “signal replacement.” Transplanting the microbiota of patients with MDD into GF or antibiotic-treated animals induces anhedonia and anxiety-like behaviors within weeks (Kelly et al., 2016). Similarly, transplantation of microbiota from donors with ASD recapitulates social deficits and stereotypic behaviors, accompanied by changes in central transcriptional and metabolic pathways (Sharon et al., 2019). Similar phenotypic transfers have been observed for obesity and AD, where recipient animals exhibit corresponding metabolic and cognitive impairments (Ridaura et al., 2013; Grabrucker et al., 2023). Critically, in genetic models of depression, supplementation with a single bacterial strain (*Akkermansia muciniphila*) reverses neuronal atrophy and depressive-like behaviors, demonstrating that microbial signals can actively reshape neural structure and behavior even in the context of fixed genetic risk (Hwang et al., 2025). This dimension establishes sufficiency.

### 3.3 Observation: multi-omics and neuroimaging map ecological association

Observational studies-large-scale human cohort analyses integrating multi-omics and neuroimaging-ask whether microbial features covary systematically with brain function in natural populations. In human studies, large-sample analyses combining multi-omics with neuroimaging have begun to map the ecological landscape of the microbiota-gut-brain axis. For example, Zhao et al. (2026) demonstrated in individuals with metabolic syndrome that specific bacterial genera correlate significantly with gray matter volume and white matter fractional anisotropy (FA). Their mediation models suggested that brain structural changes serve as a key pathway through which the microbiota influences cognitive performance (Zhao et al., 2026; Zhao et al., 2026). Furthermore, joint independent component analysis revealed that the abundances of Prevotella and Bifidobacterium correlate with the functional connectivity (FC) strength of the default mode network (DMN) and executive control network (ECN), suggesting that SCFA-producing microbiota may be involved in regulating the activity of these key brain networks (Kohn et al., 2021). Another study found that indoxyl sulfate—a gut-derived metabolite—positively correlates with anxiety levels and the FC of interoceptive regions such as the anterior insula and subgenual cortex (Brydges et al., 2021). Moreover, population-level analyses have shown that the contribution of genetics versus environment to microbiome variation is highly context-dependent, with only a minority of taxa being heritable—a finding that underscores the centrality of environmental factors, including the microbiome itself, in shaping the cognitive ecosystem (Gacesa et al., 2022). This dimension provides ecological validity.

### 3.4 Intervention: targeted remodeling of cognitive functions

Intervention studies—randomized controlled trials using probiotics, prebiotics, or dietary modifications—ask whether the cognitive system is clinically plastic and can be modulated via the microbial interface. Randomized controlled trials (RCTs) have shown that targeted interventions with probiotics (e.g., Lactobacillus plantarum DR7), prebiotics, or specific strains (e.g., *Akkermansia muciniphila*) can effectively alleviate anxiety symptoms, improve working memory, and normalize physiological indicators such as butyrate concentrations and prefrontal-amygdala connectivity (Chong et al., 2019; Higarza et al., 2021). However, emerging evidence from systematic reviews indicates that efficacy varies considerably across individuals, with host genetics and baseline microbiota composition moderating treatment response (Cho et al., 2025). This dimension confirms intervenability while highlighting the need for personalized approaches. Importantly, some studies have directly linked microbial manipulations to task-specific cognitive performance through multi-step mediation. For example, Yue et al. (2026) showed that a vagus-dependent gut microbiota–metabolite axis mediated the effect of chronic inflammatory pain on working-memory deficits, and that restoring microbial function reversed the performance impairment. Such findings provide a causal chain from microbiota to metabolite to neural pathway to cognitive task, strengthening the case for constitutive relevance.

These four dimensions are mutually complementary: causal mechanisms in animal models establish biological plausibility, systematic associations in humans demonstrate ecological relevance, and proof-of-concept interventions confirm clinical tractability. The constitutive-relevance hypothesis is strengthened by the convergence of these evidence types.

## 4 Discussion

### 4.1 Theoretical implications: expanding the 4E cognition framework

Over the past two decades, the 4E cognitive theory has continuously expanded the boundaries of traditional cognitive views. However, the “body” in existing theories is often implicitly restricted to a macroscopic biological system composed of human cells. Recent advances in microbiome research incorporate a major microbial dimension into this framework, achieving a substantive deepening of embodied cognition theory in the following four aspects:

(1) Mechanistic support for embodiment. Microbiome research provides molecular-level empirical evidence for the proposition that cognition is rooted in physiological states. Deprivation studies, using GF or antibiotic-treated models, consistently show that loss of the interoceptive signaling stream mediated by microbial metabolites (e.g., SCFAs) significantly impairs neural plasticity (Ogbonnaya et al., 2015; Sharon et al., 2019), Conversely, the reintroduction of key metabolic signals (e.g., butyrate) can rescue plasticity deficits and reverse behavioral impairments (Sun et al., 2016; Dalile et al., 2019), while rapidly modulating neural oscillations to alleviate behavioral disorders in the short term (van de Wouw et al., 2018). These strong causal experiments have advanced the “constitutive role of the body in cognition” from a philosophical construct to a mechanistic hypothesis verifiable at the biological level.

(2) Expansion of biological boundaries. Trillions of symbiotic microbes continuously regulate neural circuit function through metabolites and immune signals. Their genetic capacity, which substantially exceeds the human genome, forces a re-examination of the “body” in which cognition is embodied; it is no longer limited to human eukaryotic systems but must incorporate the symbiotic ecosystem as a functionally indispensable layer. Any embodied model that neglects the microbial dimension will omit a critical constitutive layer of the cognitive architecture. The microbiota plausibly satisfies the proposed criteria for constitutive status-spatial internality, temporal continuity, functional integration, information-processing participation, and developmental embedding—making it not merely a modulator but a constitutively relevant contributor to the interoceptive, affective, and vagal-metabolic architectures of cognition (section 1.5).

(3) A biological case for debates on the extended mind. RCTs have confirmed that reprogramming microbial metabolic functions through prebiotics, probiotics, or dietary interventions can significantly improve working memory and anxiety levels within weeks. This indicates that the microbiota can be actively harnessed as a biologically embedded resource for modulating embodied cognitive architectures, resonating deeply with the concept of the “extended mind” thesis (1998). The distinction is that this “external vehicle” is an active, biological metabolic network engaging in dynamic biochemical dialogue with the host.

(4) Philosophical and ethical frontiers of the “microbial self.” When emotional predispositions and decision-making processes are proven to be partially dependent on microbial communities, traditional categories of cognitive responsibility, personal identity, and behavioral attribution require revision. This opens up a new and urgent window for interdisciplinary dialogue between cognitive science, philosophy, and ethics.

### 4.2 Limitations and challenges

Although abundant evidence has accumulated regarding the role of the gut microbiota in embodied cognition, this field remains in its early stages and faces several fundamental challenges:

(1) Complexity of causal inference: Most current human studies are limited to cross-sectional association analyses, making it difficult to define the causal direction and temporal sequence of the “microbial molecule—host pathway—cognitive phenotype” axis. For example, while the study by Zhao et al. (2026) uncovered a microbiota–brain–cognition network through an elegant design, its cross-sectional nature precludes a definitive determination of causality (Zhao et al., 2026). Advancing from association to causation requires future research to leverage longitudinal cohorts, interventional experiments (e.g., supplementation with specific probiotics), and computational modeling to validate and visualize the complete causal chain of “strain/metabolite-cell receptor-neural circuit-behavioral output” (Zhao et al., 2026; Zhao et al., 2026).

(2) Extreme complexity and dynamic nature of the ecosystem: The gut microbial ecosystem is characterized by highly dynamism, functional redundancy, and significant inter-individual variation. Microbial composition can shift within 48 hours in response to dietary changes, yet it tends to revert to an individualized homeostasis. Furthermore, metabolic functions (e.g., SCFA production rates) vary markedly among strains of the same genus. This complexity, further shaped by host genetic and immune backgrounds, complicates the construction of universal models. Addressing these issues urgently requires the development of multi-scale ecological dynamic models and novel algorithms, such as “functional redundancy deconvolution.”

(3) Substantial gaps in mechanistic details: Although preliminary understanding of classic pathways (e.g., AhR ligands, SCFA receptors) has been achieved, a large number of low-abundance signaling molecules (e.g., specific peptides, small RNAs, and phenylpropionic acid derivatives) remain “dark matter” in the signaling landscape. Uncovering these mechanisms relies on the integration of single-cell spatial multi-omics technologies and advanced neuro-immune co-culture models.

(4) Bottlenecks in technological integration and data standardization: Synchronous collection of multimodal data generates massive information. However, the lack of unified spatiotemporal annotation and batch correction standards severely limits the reproducibility of cross-cohort studies. Promoting international cooperation to formulate FAIR (Findable, Accessible, Interoperable, Reusable) Gut-Brain metadata standards, and federated learning frameworks is crucial.

(5) Inter-individual response variability in response: Studies have found that the effects of “beneficial bacteria” are not universal. Therefore, future developments must shift towards establishing host-microbe compatibility indices and phenotype-directed probiotic libraries to achieve precise psychobiotic interventions.

(6) Controversies, reproducibility, and the challenge of confounding: The field of microbiome–brain research is characterized by substantial heterogeneity across studies-a feature that reflects the inherent complexity of the system. Large-scale human cohort studies have revealed that only a minority of microbial taxa (approximately 6.6%) are heritable, whereas nearly half of the variation is explained by co-housing environment, diet, and lifestyle (Gacesa et al., 2022). This finding tempers strong claims about genetic determinism of the microbiome and instead highlights the centrality of gene–environment interactions in shaping the microbial ecosystem.

Reproducibility remains a challenge: meta-analyses have noted inconsistent associations between specific microbial taxa and psychiatric conditions across different cohorts (Shoubridge et al., 2022). Moreover, even when associations are replicated, the directionality of causality remains contested. Mendelian randomization studies have yielded divergent results, for instance, distinct causal associations between insomnia and gut microbiota have been reported by different groups (Li Z. et al., 2023; Wang et al., 2024), underscoring the limitations of inferring causation from genetic instruments alone.

An additional layer of complexity arises from medication effects. Antipsychotics and antidepressants themselves reshape the gut microbiome, making it difficult to disentangle disease-related from drug-related microbial alterations (Li Z. et al., 2023; Simunovic Filipcic et al., 2025). This does not invalidate the constitutive hypothesis; rather, it situates the microbiota within a dynamic system where host state (including pharmacotherapy) and microbial composition co-vary.

We view these complexities as integral to the multi-level nature of the evidence base. The constitutive hypothesis is not built on the claim that microbial effects are uniform or that every association will replicate across all populations. Instead, it is supported by complementary evidence types, causal mechanisms in animals, systematic associations in humans, and proof-of-concept interventions, which together provide a more robust foundation than any single line of evidence could offer.

#### 4.2.1 Alternative interpretations: regulation, scaffolding, or constitution?

Alternative interpretations: An important alternative interpretation is that the gut microbiota regulates interoceptive and physiological states that gate or shape cognition, without thereby constituting part of cognition itself. We take this possibility seriously. Indeed, much of the current evidence most directly demonstrates that microbial signals influence inflammatory tone, stress responsivity, metabolic status, and neural plasticity—factors that condition how cognition unfolds rather than serving as canonical computational vehicles in their own right.

For this reason, we adopt a graded view. At minimum, the microbiota should not be regarded as a merely external or incidental influence, because it is continuously present, developmentally embedded, and tightly integrated with neural, immune, and endocrine processes. The strongest current case, in our view, is that the microbiota is constitutively involved in the embodied, interoceptive, and affective scaffolding of cognition. Whether some microbial contributions should further be classified as direct constituents of core information-processing operations remains an open question that requires more precise empirical and conceptual work.

This interpretation preserves the central insight of our argument—that cognition is biologically distributed across host–microbe interactions—while avoiding an overly sharp dichotomy between “mere modulation” and “full constitution.” Accordingly, we propose that the gut microbiota is best understood as a constitutively relevant contributor to interoceptive, affective, and vagal-metabolic architectures—not as a universal part of all cognitive processes.

#### 4.2.2 Falsifiability and empirical adjudication

##### 4.2.2.1 Falsifiability

Our constitutive claim is not unfalsifiable. The weaker “scaffolding” or “modulatory” interpretation would be favored if future studies consistently showed that microbial effects on cognition are fully mediated by non-specific changes in inflammation, stress hormones, or global arousal, without evidence for process-specific online participation. Concretely, if selective vagal blockade or targeted receptor antagonists (e.g., FFAR2/3, AhR) failed to abolish microbe-to-cognition effects, or if microbial manipulations influenced cognitive performance only when combined with drastic changes in diet or metabolic state, the case for constitutive relevance would weaken. Conversely, demonstrating that microbial metabolites directly modulate task-related neural activity in a pathway-specific manner, independent of general state changes, would further support our claim. We present this falsification criterion to make our position transparent and to guide future experimental tests.

## 5 Conclusion and future perspectives

Through theoretical elaboration and comprehensive analysis of multi-layered empirical evidence, this paper establishes that the gut microbiota is an indispensable and deeply embedded biological dimension of embodied cognition, acting as a constitutively relevant contributor to the interoceptive, affective, and vagal-metabolic architectures that help organize specific cognitive processes and shape how they unfold. As a large-scale, active signaling network, it continuously produces a variety of bioactive molecules that shape interoceptive states, emotional response thresholds, decision-making preferences, and even social interaction patterns across different time scales—from milliseconds to days—via neural, immune, and endocrine pathways.

Re-examining gut-brain communication from the perspective of embodied cognition represents an important paradigm shift: cognition is no longer confined within the skull but is understood as being embedded in a cross-species, ecological biological system. In this system, the host and its symbiotic microbes continuously exchange signals across multiple physiological interfaces, giving rise to emotional states, decision-making, and social behavior. This transformation opens up new avenues for brain health maintenance and psychiatric disorder intervention. Strategies such as targeting the early window of microbial colonization, developing personalized psychobiotics, and designing targeted metabolic mimetics gradually emerge as novel, programmable precision interventions in translational medicine.

To truly realize the potential of this field and translate scientific insights into human well-being, a profound interdisciplinary collaborative effort is essential, focusing on the following key future directions:

(1) Paradigm innovation in mechanism elucidation: Future research must prioritize the integration of humanized organoids, targeted colonization of GF animals, and in situ real-time imaging to achieve whole-chain causal visualization of “strain/metabolite—cell-specific receptor—neural circuit dynamics—behavioral output.” The application of CRISPR-based in vivo tracking and optogenetic functional blockade will provide precise experimental paradigms to verify the causal roles of low-abundance signaling molecules.

(2) Computational models and digital twins. Unraveling the high dynamism and inter-individual variability of the gut ecosystem relies on constructing cross-scale dynamic causal networks integrating metagenomics, metabolomics, neuroimaging, and behavioral data. Introducing digital twin technology to build personalized virtual microbiota-brain models and simulating long-term interventions *in silico* will provide predictive blueprints for individualized clinical strategies.

(3) Precision engineering of intervention strategies. Future microbial interventions must move beyond the “one-strain-fits-all” paradigm toward personalized regimens based on an individual’s “microbiota-metabotype” functional fingerprint. Meanwhile, exploring small-molecule “metabolic mimetics” and CRISPR-precisely edited engineered strains will define the frontier of next-generation psychobiotics.

(4) Lifespan perspective and big data integration. Establishing large-scale longitudinal cohorts (e.g., 3,000 participants from the perinatal period to old age), with bi-annual collection of multi-omics and fMRI data is essential. Using cross-lagged panel models to identify critical microbial windows affecting cognitive development and promoting international cooperation to formulate FAIR Gut-Brain metadata standards will be key to maximizing data value.

(5) Exploring social cognition and the “collective mind.” Adopting innovative experimental designs such as dyadic fMRI and group-synchronized EEG to investigate the embodied mechanisms of symbiotic microbes in advanced social behaviors (e.g., empathy, trust, cooperation) will provide a novel microbiological explanation for the “collective distributed mind.”

(6) Constructing a unified “microbiota-mind” model and ethical framework. We define the microbiota-mind model as a conceptual framework that integrates microbial ecology, neural computation, and cognitive phenomenology to explain how mental processes emerge from dynamic host–microbe interactions. Ultimately, we need to establish a predictable and intervenable unified model across ecological, molecular, neural, and behavioral scales.

Translational considerations: from proof-of-concept to clinical practice. The promise of microbiota-based interventions for cognitive and emotional disorders requires consideration of the realities of clinical translation. Animal models offer compelling proof of concept: targeted microbial interventions reverse genetically driven neuronal atrophy and behavioral deficits (Davidson et al., 2024; Hwang et al., 2025).

In humans, however, the microbiome is embedded in a complex web of environmental and genetic factors, diet, medication, socioeconomic status, early-life exposures, and host genetics all shape microbial composition (Gacesa et al., 2022). As a result, the efficacy of any given probiotic strain varies across individuals, a finding increasingly recognized in precision nutrition and psychiatry (Cao et al., 2025). Moreover, psychiatric medications themselves remodel the gut microbiome, creating bidirectional feedback loops that complicate intervention design (Simunovic Filipcic et al., 2025).

These complexities refine rather than diminish the translational potential of microbiome-targeted strategies. Moving forward requires: (1) biomarkers that predict individual response, including host genetic variants and baseline microbial profiles; (2) standardized protocols to enable replication across trials; (3) integration of microbiome interventions with existing therapies; and (4) longitudinal studies to establish long-term safety and durability. A cautious optimism is warranted—one that acknowledges the gap between controlled experimental settings and real-world complexity, while recognizing the microbiota as a uniquely accessible, programmable interface for modulating cognitive function from the periphery.

By advancing across these six dimensions, gut-brain embodied research is expected to form a multi-dimensional scientific system spanning from basic mechanisms to clinical applications, and from the individual biological processes to population-level patterns. This synthesis will not only transform profound philosophical reflections into tangible improvements in human well-being but may ultimately resolve a fundamental question: in the broad landscape of life and cognition, how do humans and their symbiotic microbial communities jointly shape the embodied conditions under which subjectivity and cognition emerge?
